# Association of hormone receptors and human epidermal growth factor receptor-2/neu expressions with clinicopathologic factors of breast carcinoma: a cross-sectional study in a tertiary care hospital, Kabul, Afghanistan

**DOI:** 10.1186/s12885-024-12129-5

**Published:** 2024-03-27

**Authors:** Esmatullah Esmat, Ahmed Maseh Haidary, Ramin Saadaat, Syeda Naghma Rizvi, Syeda Aleena, Mujtaba Haidari, Sayed Murtaza Sadat Hofiani, Nasrin Hussaini, Ahmadullah Hakimi, Abdullatif Khairy, Jamshid Abdul-Ghafar

**Affiliations:** 1 Department of Pathology and Clinical Laboratory, French Medical Institute for Mother and Children (FMIC), Kabul, Afghanistan; 2 Department of Academic and Research, Postgraduate Medical Education (PGME), French Medical Institute for Mothers and Children (FMIC), Kabul, Afghanistan; 3 Aga Khan University School of Nursing and Midwifery (AKU-SoNaM), Karachi, Pakistan

**Keywords:** Breast cancer, Estrogen receptor, Her2/ neu receptor, Afghanistan, Pathological factor

## Abstract

**Background:**

Breast cancer (BC) is one of the major causes of death worldwide. It is the most common cause of death before the age of 70 years. The incidence and mortality of BC are rapidly increasing, posing great challenges to the health system and economy of every nation.

**Methodology:**

A cross-sectional analytical study was conducted at the Department of Pathology and Clinical Laboratory of the French Medical Institute for Mothers and Children (FMIC) to demonstrate the association of human epidermal growth factor receptor 2 (Her2/Neu) and estrogen receptor (ER)/ progesterone receptor (PR) with clinical as well as pathological parameters among women with BC. A consecutive nonprobability sampling method was used for this study over a span of one and a half years.

**Results:**

One hundred twenty participants diagnosed with breast cancer were included in the study. The mean age at diagnosis was 44.58 ± 11.16 years. Out of the total patients, 68 (56.7%) were above 40 years old, 108 (90%) were married, 94 (78.3%) were multiparous, and 88 (73.3%) had a history of breastfeeding. 33.3% of cases were within the age range of menopause (40–50 years). The positive expression rates of ER, PR, and Her2/neu were found to be 48.8%, 44.6%, and 44.6%, respectively, and Her2/neu overexpression was found to be higher among ER/PR-negative cases.

**Conclusion:**

In our study, we demonstrated that among Afghan women, grade II invasive ductal carcinoma, not otherwise specified, was the most common type of BC and frequently affected women above the age of 40. We also revealed that the percentage of negative ER (50.4%), negative PR (54.4%), and concordant ER/PR-negative cases were high compared to other possibilities. Additionally, the study revealed that expression of Her2/neu was in contrast with the expression of ER and PR receptors. The findings of our study still support the importance of performing immunohistochemical stains for hormonal receptor classification in terms of better clinical outcomes and prognosis.

## Background

Breast cancer (BC) is the most common malignancy affecting women globally [1]. According to the World Health Organization’s (WHO) estimation in 2020, BC had the highest prevalence rate of 2.3 million cases (11.7%), resulting in 6.9% deaths. BC is not a single disease but a group of several important tumor subtypes, each with different natural characteristics that require different management strategies [1]. Invasive ductal carcinoma (IDC), not otherwise specified (NOS), accounts for the highest number of BC cases (75–80%) [2]. BC is responsible for over one million of the 10 million neoplasms diagnosed worldwide each year [3]. In 2018, out of 19,450 cancers diagnosed in all age groups and both sexes in Afghanistan, 3,062 were BC, according to WHO data from neighboring countries [4].

The burden of BC can be reduced by early detection and diagnosis. Mammography is the gold standard technique for detecting early breast carcinoma, specifically the ductal carcinoma, even before clinical signs and symptoms appear. By using mammography, the mortality rate has decreased up to 25%-30% [5]. Fine Needle Aspiration Cytology (FNAC) is the least invasive method of biopsy without leaving any scar [6]. Core needle biopsy is another sampling method used with larger hollow needle than fine needle aspiration. Usually, core needle biopsy does not leave scar. Both techniques are used and offer quick results without significant discomfort and scarring.

Incisional biopsy is like regular surgery which is more invasive than needle biopsy [7]. It leaves scar, takes more time to recover and most importantly suboptimal for staging of BC [7]. Lumpectomy, mastectomy or radical mastectomy are the biopsy methods which attempts to remove the entire area of suspicious tissue from the breast along with normal area or entire breast with axillary lymph nodes.

The above-mentioned biopsy methods and tests used for screening and diagnosis of BC are not sufficient for definite diagnosis. Other advanced tests, such as identification of genetic, hormonal, and growth factor receptors, are necessary. Genetic testing is a powerful tool that allows for the detection of the breast cancer genes (BRCA 1 and BRCA 2), and non-BRCA germline mutations in individuals at high risk of BC. Similarly, immunohistochemical (IHC) markers of prognostic significance that are frequently used for BC are estrogen receptor (ER), progesterone receptor (PR), and human epidermal growth factor receptor 2 (Her2/neu). Estrogen and Progesterone are the two most important steroid hormones in the female body responsible for various female physiological and physical characteristics. They are involved in regulating the menstrual cycle and play an important role in pregnancy. Similarly, the growth of breast organs depends on the concentration of estrogen and progesterone. BC with ER and PR positivity have a good prognosis and show a lower risk of mortality [8]. The Her2/neu receptor is a protein in the human body encoded by the erythroblast oncogene B2 (ERBB2) gene located on (17q12). First derived from rodent glioblastoma, Her2/neu or p185 is present in human normal cells and is necessary for cellular growth. Overexpression of this gene is responsible for uncontrolled cell growth, rapid tumor growth, and lower survival rates. However, its advantage is good response to Herceptin therapy. Therefore, understanding the collaboration between Her2/neu polymorphism in BC can be effective in determining treatment strategies and evaluating prognosis. Overexpression of HER2/Neu can also be found in endometrial, gastric, and prostate cancer, but its positivity is lower than BC [9, 10].

Several studies have investigated the association between the histopathology of breast tumors, including tumor grade and type, and IHC markers such as ER, PR, and HER2 receptors [11]. It has been found that there are multiple risk factors for BC that are associated with ER and PR [12]. However, there is currently inadequate evidence about the prognostic effects of these receptors in different types of BC in developing countries [8]. In addition, the association of these important hormones with other risk factors for BC has not been studied in Afghanistan. This current research attempts to provide information about the profile of hormonal receptors in BC in Afghanistan, and to the best of our knowledge, this is the first study to demonstrate the association of clinical characteristics of BC with the status of ER, PR, and HER2 in Afghanistan.

## Methodology

A cross-sectional analytical study was conducted at the Department of Pathology and Clinical Laboratory of the French Medical Institute for Mothers and Children (FMIC), the only diagnostic center in Afghanistan capable of performing IHC staining for definitive diagnosis, tumor typing, and evaluation of ER/PR, Her2/neu, and other prognostic tumor markers. The study population included females whose breast samples were received at the histopathology section from December 1, 2020, to March 31, 2022, for testing Her2/neu and ER, PR status in 20- 80 years of age group and they meet the inclusion criteria of the study. A total of 120 cases were collected. Permission was obtained from FMIC’s Ethical Research Committee (ERC) and the Institutional Review Board (IRB) of the Ministry of Public Health (MoPH) of Afghanistan. The identity of the participants was not disclosed, and they were given numeric codes. The consent form was signed by the patient or their relative who brought the sample after obtaining the patient’s agreement.

The study includes all female breast samples received for Her2/neu and ER, PR status testing. The inclusion criteria involve individuals aged 20–80 who agreed to participate in the study. Breast sample received for any other histopathologic evaluation other than Her2/neu and ER, PR, male breast samples, patients not responding to telephone calls, those whom did not want to participate in the study and the breast samples that did not meet the inclusion criteria were excluded. Consecutive non-probability sampling was used for this study. Data was collected from reports of histopathology samples for Her2/neu and ER, PR status testing through the Integrated Laboratory Management System (ILMS). Other information such as marital status, parity, number of children, history of breast feeding, family history and residence were collected by phone call from patients.

Data collection form designed as a questionnaire which included both dependent and independent variables such as age of patient, marital status, parity, number of children, history of breast feeding, family history, residence tumor grade, tumor size and tumor histological types. The portion of the data, which was not available on ILMS, was collected through telephone calls such as marital status, parity, number of children, history of breast-feeding, family history and residence.

Data were analyzed by Statistical Package for Social Science (SPSS) software version 25. Descriptive statistics was used to estimate the expression rates of ER, PR and Her2/neu receptors.

Continuous variables were summarized with means and Standard Deviations (SD). Frequencies and percentages were used for categorical variables. Frequencies, percentages, means and standard deviations obtained as appropriate. The association between IHC stains and clinic-pathological characteristics was assessed by using Pearson Chi-square test. The *p* value less than 0.05 was taken as significant.

**Fig. 1:**
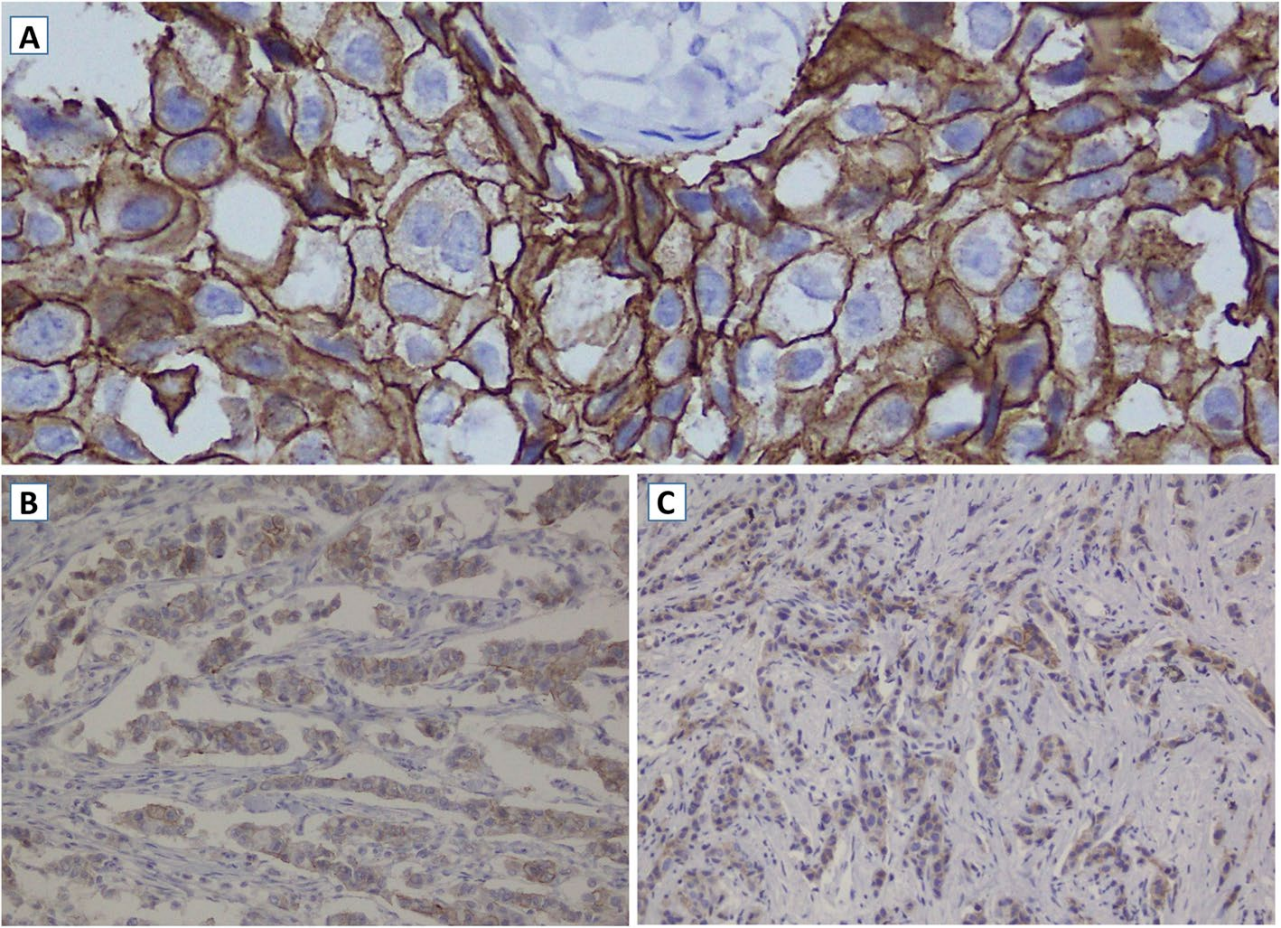
**A** Her2 IHC stains showing more than 10% complete membranous positivity (score + 3), **B** Her2 IHC stains showing weak to moderate complete membrane staining observed in more than 10% of tumor cells (score 2 +), **C** Incomplete membrane staining and within more than 10% of tumor cells (score 1 +)

**Fig. 2:**
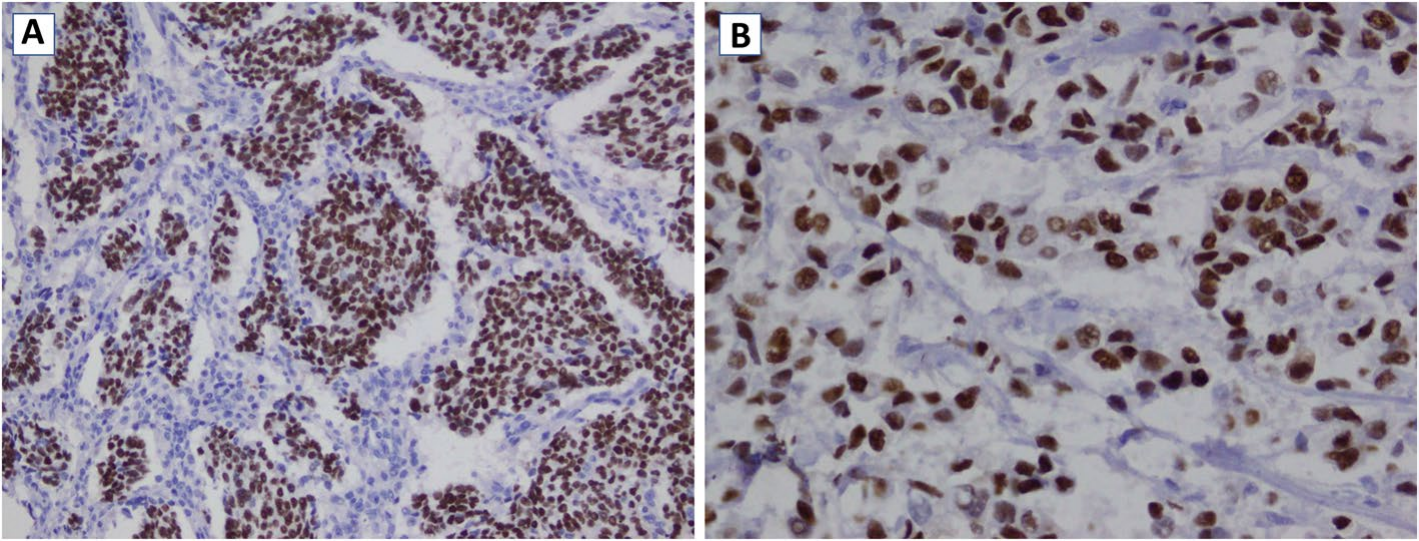
**A** ER IHC stains showing nuclear positivity in tumor cells, **B** PR IHC stains showing nuclear positivity in tumor cells

The IHC stains were performed according standard protocol and automated auto-Stainer machine (Autostainer 480S REF # A80500007, Thermo scientific) was used for this purpose. It is a chromogen method that includes several steps including antigen retrieval, blocking, primary antibody, chromogen application and counter stain as per the protocol [13, 14]. The positivity was graded as per Collage of American Pathologist (CAP) template for reporting results of biomarker testing [15]. All results are examined by histopathologist and reviewed and finalized by a senior pathologist. And tumor cells show more than 10% strong complete membranous staining for Her2/neu consider as scoring (+ 3) or over expiration and score (0) and score (+ 1) consider negative and score (+ 2) considered as equivocal result. The intensity of membranous staining was compared with internal quality control [15, 16] (Fig. 1). More than 1% nuclear positivity ER and PR considered as positive result. The percentage of positivity were reported as (1–10%), (11–20%), (21–30%), (31–40%), (41–50%), (51–60%), (61–70%), (71–80%), (81–90%) and (91–100%) [15]. The average intensity of nuclear positivity compere to internal control was reported weak, moderate and strong [15, 17] (Fig. 2). The tumor histological grade as grade 1, grade 2 and grade 3 were determined for each individual case according to Nottingham modification of the Bloom and Richardson Grading System) [18].

## Result

Table 1 shows that the study included 120 primary BC cases, all of whom were female. The mean age of the patients was 44.58 ± 11.16 years. Of these patients, 68 (56.7%) were above 40 years old, and 108 (90%) were married. Among married women, 94 (78.3%) were multiparous, and 88 (73.3%) had a history of breastfeeding. Less than forty percent of women (40, 33.3%) were within the age range of menopause (40–50). Almost half of the sampled population had Pashtun ethnicity (54, 50%), followed by Tajik (48, 40%), Hazara (7, 5.8%), and Uzbek (7, 5.8%) ethnicities. Most of the women lived in urban areas (82, 68.3%) as compared to rural areas (38, 31.7%). Of the 120 patients, 76 (80%) had no history of BC in their first-degree relatives.

**Table 1:** Clinicopathological profile of breast carcinoma patients

Parameters	N^a^ (%)
**Marital status**	
Married	108 (90)
Unmarried	12 (10)
**Age category**	
≤ 39	52 (43.3)
≥ 40	68 (56.7)
**Parity**	
Multiparous	94 (78.3)
Nulliparous	6 (5)
Infertile	7 (5.8)
Not Applicable	13 (10.8)
**Number of children**	
1 to 3	29 (24)
4 to 5	33 (27.3)
> 6	34 (28.1)
Not Applicable	24 (19.8)
**History of breast feeding**	
Yes	88 (73.3)
No	10 (8.3)
No Applicable	22 (18.3)
**Age in menopause**	
< 40 years	4 (3.3)
40–50 years	40 (33.3)
> 50 years	12 (10)
No menopause	64 (53.3)
**Ethnicity**	
Pashtun	54 (50.0)
Tajik	48 (40)
Hazara	7 (5.8)
Uzbek/ Turkmen	7 (5.8)
Others	4 (3.3)
**Residential area**	
Urban	82 (68.3)
Rural	38 (31.7)
**Family history breast Cancer**	
Yes	29 (24.0)
No	91 (75.2)
**Family history of first degree relative**	
Yes	19 (20)
No	76 (80)
**Tumor size category**	
< 2	6 (5.0)
2 – 5	44 (36.7)
> 5	16 (13.3)
Not Applicable	54 (45.0)
Size was unknown^b^	39 (41.1)
**Histological grade**	
Grade I	3 (2.5)
Grade II	65 (54.2)
Grade III	44 (36.7)
Not graded	8 (6.7)
**Histological type**	
Invasive ductal carcinoma	110 (91.7)
Lobular carcinoma	5 (4.2)
Apocrine carcinoma	3 (2.5)
Others	2 (1.7)
**Age at menarche**	
< 12 Year	49 (40.8)
12–13 year	49 (40.8)
> 13 year	18 (15)
Unknown	4 (3.3)
**Tumor side**	
Right	60(50)
Left	58(48.3)
Bilateral	2(1.6)

^a^Number of patients

^b^Size of the tumor was unknown

All patients with BC were categorized according to their histological type. The results showed that the most common form of cancer was IDC, NOS, constituting 110 cases (91%), followed by 5 cases (4.2%) of lobular carcinoma, and only a few patients were found to have apocrine carcinoma, constituting only 3 cases (2.5%). The most common histological tumor grade was grade 2, constituting 65 cases (54.2%), followed by 44 cases (36.7%) that were grade 3, and only 3 cases (2.5%) had grade 1 tumors.

Tumor size was divided into three categories: category 1 (tumor size ≤ 2 cm), category 2 (2–5 cm), and category 3 (≥ 5 cm) in diameter. 44 cases (36.7%) had a tumor size ranging from 2–5 cm. Similarly, the location of the tumor was observed on three sides: right breast, left breast, and bilateral. The most prevalent side of the tumor observed was the right breast, constituting 60 (50%) cases, as compared to 58 cases (48.3%) that were on the left breast (Table 1).

**Fig. 3:**
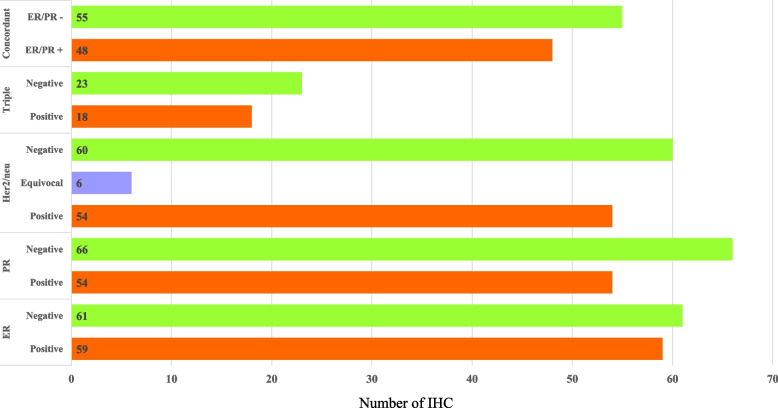
IHC profile of the study group

As shown in Fig. 1, 59 (48.8%) cases were ER-positive, and 61 (50.4%) were ER-negative. Similarly, 54 (45.3%) cases were PR-positive, and 66 (54.5%) cases were PR-negative. The ER-negative expression rate was higher than the ER-positive, as was the case with PR. Likewise, 54 (44.6%) cases were HER2/neu-positive, and 60 (49.6%) were HER2/neu-negative. Triple-negative expression for ER, PR, and HER2/neu was observed in 23 (19%) cases, while 18 (14.9%) cases were triple-positive for the expression of ER, PR, and HER2/neu. Concordant cases in which both ER and PR showed similar results, i.e., either both positive or both negative, were observed in 48 (39.7%) positive cases and 55 (45.5%) negative cases (Fig. 3).

Table 2 shows the associations of ER, PR, and HER2/neu with clinicopathological factors. Married women were observed to have higher expression rates of positive ER cases as compared to unmarried women, constituting 50% and 41% of the cases, respectively. Similarly, for positive PR, 45.3% of cases were positive, and 41.6% of cases were negative. Considering the expression of HER2/neu, it was also found to be lower among married women as compared to unmarried women, with each category constituting 43.5% and 58.3%, respectively. However, no significant association was found between marital status and ER, PR, and HER2/neu, with insignificant *p* values greater than 0.05 for each receptor, respectively.

**Table 2:** Association of ER, PR and Her2/neu receptor expression with different clinicopathological factors

Parameters	ER statues N (%)			PR statues N (%)			Her2/neu statues N (%)			
	**ER + **	**ER-**	***P* value**	**PR + **	**PR-**	***P* value**	**Her2 + **	**Her2-**	**Equivocal**	***P* Value**
	**59 (48.8)**	**61 (50.4)**		**54 (44.6)**	**66 (54.4)**		**54 (44.6)**	**60 (49.6)**	**6 (5.0)**	
**Marital status**										
**Married**	54 (50)	54 (50)	0.584	49 (45.3)	59 (54.6)	0.807	47 (43.5)	56 (51.8)	5 (4.6)	0.458
**Unmarried**	5 (41.6)	7 (58.3)		5 (41.6)	7 (58.3)		7 (58.3)	4 (3.7)	1 (0.9)	
**Age category**										
**≤ 39**	26 (50)	26 (50)	0.873	24 (44.4)	28 (55.5)	0.824	20 (37.0)	28 (46.6)	4 (66.6)	0.29
**≥ 40**	33 (50.7)	35 (49.2)		30 (44.1)	38 (55.8)		34 (62.9)	32 (53.3)	2 (33.3)	
**Parity**										
**Multiparous**	50 (53.1)	44 (46.8)	0.219	46 (48.9)	48 (51.0)	0.183	41 (43.6)	49 (52.1)	4 (4.2)	0.755
**Nulliparous**	2 (33.3)	4 (66.6)		1 (16.6)	5 (83.3)		3 (50)	2 (33.3)	1 (16.6)	
**Infertile**	1 (14.3)	6 (85.7)		1 (14.3)	6 (85.7)		3 (42.8)	4 (51.1)	0	
**Not applicable**	6 (46.1)	7 (53.8)		6 (46.1)	7 (53.8)		7 (53.8)	5 (38.4)	1 (7.6)	
**History breast feeding**										
**Yes**	46 (52.2)	42 (47.7)	0.598	42 (47.7)	46 (52.2)	0.304	41 (46.5)	43 (48.8)	4 (4.5)	0.838
**No**	5 (50)	5 (50)		4 (40)	6 (60)		3 (30)	6 (60)	1 (10)	
**Not Applicable**	8 (36.3)	14 (63.6)		8 (36.3)	14 (63.6)		10 (45.4)	11 (50)	1 (4.5)	
**Age in menopause**										
**< 40 years**	2 (50)	2 (50)	0.269	2 (50)	2 (50)	0.148	0	4 (100)	0	0.428
**40–50 years**	17 (42.5)	23 (57.5)		15 (37.5)	25 (62.5)		17 (42.5)	21 (52.5)	2 (5.0)	
**> 50 years**	9 (75)	3 (25)		9 (75)	3 (25)		5 (41.6)	7 (58.3)	0	
**Not in menopause**	31 (48.4)	33 (51.5)		28 (43.7)	36 (56.2)		32 (50)	28 (43.7)	4 (6.2)	
**Postmenopausal**	28 (49.1)	29 (50.8)		26 (45.6)	31 (54.3)		23 (40.3)	32 (56.1)	2 (3.5)	
**Ethnicity**										
**Pashtun**	24 (44.4)	30 (55.6)	0.494	26 (48.1)	28 (51.8)	0.962	25 (56.8)	18 (40.9)	1 (2.2)	0.028
**Tajik**	28 (58.3)	20 (41.6)		21 (43.5)	27 (56.5)		17 (38.6)	26 (59)	5 (11.3)	
**Hazara**	2 (28.5)	5 (71.4)		2 (28.5)	5 (71.4)		1 (14.2)	6 (85.7)	0	
**Uzbek/ Turkmen**	3 (42.8)	4 (57.1)		3 (42.8)	4 (57.1)		5 (71.4)	2 (28.6)	0	
**Others**	2 (50)	2 (50)		2 (50)	2 (50)		2 (50)	2 (50)	0	
**Residential area**										
**Urban**	45 (76.2)	14 (23.8)	0.06	39 (47.5)	43 (52.4)	0.407	37 (45.1)	39 (47.5)	6 (7.3)	0.559
**Rural**	37 (60.6)	24 (39.3)		15 (39.4)	23 (60.5)		23 (60.5)	15 (39.5)	0	
**Family history breast cancer**										
**Yes**	16(55.1)	13 (44.8)	0.459	15 (51.7)	14 (48.2)	0.403	17 (58.6)	11 (37.9)	1 (3.4)	0.555
**No**	43 (53.0)	38 (46.9)		29 (56.8)	52 (43.1)		43 (47.2)	43 (47.2)	5 (5.4)	
**Tumor size category**										
**< 2 cm**	2 (33.3)	4 (66.6)	**0.047**	1 (16.6)	5 (83.3)	**0.021**	2 (30)	4 (60)	0	0.883
**2-5 cm**	29 (65.9)	15 (34.0)		27 (69.2)	12 (30.7)		12 (33.3)	23 (63.8)	1 (2.7)	
**> 5 cm**	6 (37.5)	10 (62.5)		4 (19.0)	17 (80.9)		7 (43.7)	8 (50)	1 (6.2)	
**NA**^**a**^	22 (40.7)	32 (59.2)		22 (40.7)	32 (59.2)		25 (46.2)	25 (46.2)	4 (7.4)	
**Histological grade**										
**Grade I**	3 (100)	0	0.014	3 (100)	0	0.105	0	3 (100)	0	0.021
**Grade II**	38 (58.4)	27 (41.5)		32 (49.2)	33 (50.7)		22 (33.8)	40 (61.5)	3 (4.6)	
**Grade III**	14 (31.8)	30 (68.1)		17 (38.6)	27 (61.3)		28 (63.6)	14 (31.8)	2 (4.5)	
**Not graded**	4 (50)	4 (50)		2 (25)	6 (75)		4 (50)	3 (37.5)	1 (12.5)	
**Histological type**										
**IDC-NOS**	53 (48.1)	57 (51.8)	0.059	49 (44.5)	61 (55.4)	0.232	48 (43.6)	57 (51.8)	5 (4.5)	0.388
**Lobular carcinoma**	5 (100)	0		4 (80)	1 (20)		2 (40)	2 (40)	1 (20)	
**Apocrine carcinoma**	1 (100)	0		1 (33.3)	2 (66.6)		3 (100)	0	0	
**Others**	0	2 (100)		0	2 (100)		1 (50)	1 (50)	0	
**Age at menarche**										
**< 12 Year**	23 (46.5)	26 (53.4)	0.095	20 (40.8)	29 (59.1)	0.5	27 (55.1)	21 (42.8)	1 (2.0)	0.14
**12–13 year**	20 (40)	29 (60)		21 (42.8)	28 (57.1)		20 (40.8)	25 (51.0)	4 (8.1)	
**> 13 year**	13 (69.2)	5 (30.7)		11 (61.1)	7 (38.8)		6 (33.3)	12 (66.6)	0	
**Unknown**	3 (75)	1 (25)		2 (50)	2 (50)		2 (40)	2 (40)	1 (20)	
**Tumor side**										
**Right**	30 (50)	30 (50)	0.982	29 (48.3)	31 (51.6)	0.742	22 (36.6)	36 (60)	2 (3.3)	0.149
**Left**	28 (48.2)	30 (51.7)		24 (41.3)	34 (58.6)		30 (51.7)	24 (41.3)	4 (6.8)	
**Bilateral**	1 (50)	1 (50)		1 (50)	1 (50)		2 (100)	0	0	

*N* Number of cases

^a^Not applicable

Women were categorized into four age ranges in which they usually start experiencing menopause symptoms: less than 40 years, between 40 and 50 years, above 50 years, and no menopausal age. The data shows that women above 50 years of age had significantly higher positive expression rates for estrogen receptors (75%) than women less than 40 years of age (50%). Similar significant associations were observed between progesterone receptor (75%) and age at menopause. The data also showed higher positive overexpression rates for Her2/neu for older women, and the association was significantly observed (41.6% vs. 0%) between Her2/neu and age at menopause less than 40 years.

Women from urban settings showed higher expression rates of ER (76% vs. 60.6%), PR (47.5% vs. 39.4%), and Her2/neu (45.1% vs. 60.5%) than women living in rural settings, but none showed a statistically significant association. The results showed relatively higher positive expression rates of Her2/neu receptors among Uzbeks than Pashtun and other ethnicities. However, for ER and PR, Tajik and Pashtun women had higher positive expression rates than women from other ethnicities. Yet, the difference was not statistically significant between ethnicity and receptors.

The data showed that women who had a family history of BC had higher positive expression rates for ER and Her2/neu receptors; ER (55.1% vs. 53.0%) and Her2/neu (58.6% vs. 47.2%) compared to women with no history in the family. But no significant difference was observed.

The positive expression rates of ER (65.9% vs. 34%) and PR (69.2% vs. 30.7%) were significantly higher among women whose tumor size was 2 – 5 cm than women who tested negative on hormone receptors. Overexpression of Her2/neu was higher for patients whose tumor size was greater than 5 cm than patients with a tumor size of 2 – 5 cm and less than 2 cm. The difference was not found significant. ER and PR positive expression rates were very high for patients diagnosed with lobular carcinoma and apocrine carcinoma compared to patients diagnosed with IDC, NOS. But the association of histopathological type and receptors was found to be non-significant. Women with grade 1 and 2 carcinomas showed higher positive expression rates for ER and PR than Her2/neu receptors. However, cases with grade 3 of cancer showed higher positive rates for Her2/neu than other receptors. The side of the tumor did not show any significant association with receptors as well (Table 2).

**Fig. 4:**
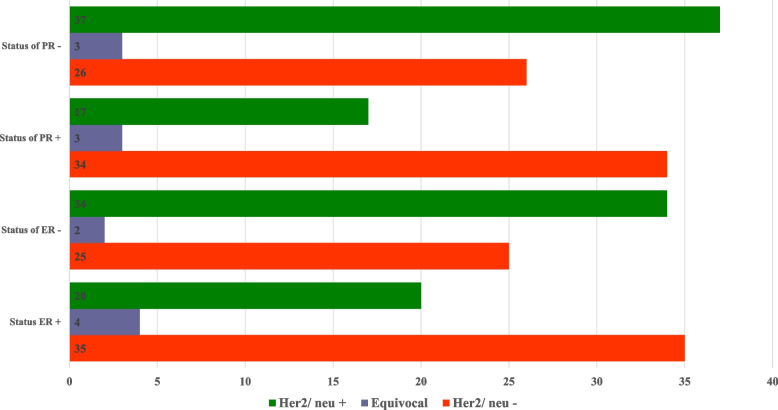
Correlation of ER, PR and HER2/neu

Figure 2 shows the association of Her2/neu with ER & PR. The cross-tabulation showed that 120 patients were tested for Her2/neu receptors, and out of them, 54 (44.6%) were positive. Among those who tested positive, 20 (37%) were found to be ER positive and 17 (31.8%) were PR positive. However, no statistical difference was found between Her2/neu and ER & PR, respectively. Hence, the results concluded that overexpression of Her2/neu was higher among patients who tested negative on estrogen and progesterone (Fig. 4).

## Discussion

The aim of our study was to demonstrate the association of ER, PR and Her2/neu receptors expression in BC with other significant clinicopathological factors among Afghan women. In this study the mean age at diagnosis was 44.73 range of (22–75). Mean ages were reported by similar study in Bangladesh [19], Qatar [20], Pakistan [21] and Iran [8]. These statistics indicate that the prevalence of breast carcinoma in our population is among the younger women as compared to other countries. It could be due to prolonged war in the country, stress of war, life threating situation, negative psychological effect, environmental pollution and other genetic factors [22–24]. Most of the old age women with BC die before diagnosis due to unavailability of a standard health care system and poor economy in the country. Parity and lactation has similar protective effects on BC [25]. In this study, most of the women 94 (78.3%) were multiparous and had history of breast feeding in 88 (73.3%) cases. Early menarche and late menopause are well known risk factors for BC among postmenopausal women. Younger age at menarche is related to earlier onset of regular menstrual cycles, early hormonal exposure due to regular ovulatory menstrual cycles may be an important etiologic factor. In addition, women with younger age at menarche have higher estrogen levels for multiple years throughout their reproductive lives [26]. In the present study 49 (40.8%) BC patient has experienced her first menorrhea before the age of 12 years, From 52 younger women (age group < 39 years) 26 of them had experienced their first menarche before age of 12 years and 12 (10%) patients experienced menopause at > 50 years of age.

One of the important risk factors for BC was family history of BC in the first degree relatives. The role of BRCA gene mutation in BR has been studied in different studies that demonstrated approximately 11 times more risk to develop BC for women who harbor the mutation [27]. 55%—72% of women who inherit BRCA1 gene will experience BC as per national cancer institute [28]. In present study 19 (20%) patients had a history of first degree relative diagnosed with BC, somewhere during their lifetime.

Tumor size is one the essential elements of pathological staging of BC, as there is a direct relation with tumor size and metastasis of the tumor in axillary lymph nodes, poor survival [29], prognosis, and therefore the treatment plan. In this study the majority of samples received as core needle biopsy (45%), which were not possible to record the exact size of the tumor. Considering the modified Bloom—Richardson grading system or Nottingham grading system, our study showed that majority of tumor including 110 (91.7%) patients were IDC, NOS, 65 (54.2%) were grade 2 and 44 (36.7%) were intermediate size (2–5 cm), which is consistent with the result of Cherry Bansal found in Indian papulation in which 56.2% of the tumor were intermediate in size (2–5 cm) and 64% were IDC, NOS grade 2 [30]. There is a relation between grade of tumor and metastasis to axillary lymph nodes, high-grade tumors have more sentential lymph node metastasis [31, 32]. Multiple clinicopathologic factors affect prognosis and therefore the outcome of management in patients with BC, including histological type, grade, stage, status of ER, PR and Her2/neu, BRCA 1 status [33, 34]. Since BC is heterogeneous, many prognostic and predictive markers have been proposed to determine and provide information about a patient’s response to treatment. Predictive markers provide information regarding patient response to treatment, whereas a prognostic marker indicates the overall survival of the patient regardless of the therapy. Her2/neu-positive BC has been demonstrated to have different molecular and clinical features. Overexpression of Her2/neu has an adverse prognostic effect that is associated with poorly differentiated, high-grade tumors, high rates of cell proliferation and a relative resistance to certain types of chemotherapy, have worse prognosis and axillary lymph node metastasis [16, 35, 36].

In the present study we found significant association of Her2/neu with histological grade (*p* = 0.021) which was more positive in high grade tumors than low grade tumors. Alike relation but not significant reported by Fouzia Shaikh [37] and Pritu Yadav [38] who revealed that high grade tumors were positive for Her2/neu. Moreover other literatures also show association of high grade tumors with Her2/neu positivity [37–40]. However, there are some studies that showed significant correlation between Her2/neu and histological grade (high grade) of tumor but are Her2/neu negative [37]. Although this study did not report significant association of Her2/neu overexpression with ER (*p* = 0.236) and PR (*p* = 0.162) positivity, there was reverse relationship between them, this relationship showed that overexpression of Her2/neu which is negative for ER and PR. The same result with significant *p* value found in the other studies Edlira Pajenga (*p* = 0.02) [41] and H J Huang (*p* = 0.001) [42], Hussain Gadelkarim Ahmed and other studies [39, 40, 43] which support the hypothesis of reverse association of ER with Her2/neu. The major difference between the studies with significant *p* value is because of the sample size; they performed the study on a larger sample (Edlira Pajenga with 123 sample and H J Huang with 1688 samples).

Expression of ER and/PR is a very important prognostic and predictive factor and indicates a better outcome as compared to tumors with negative status. Accessary hormonal therapy is favor for all the women with positive ER/PR status regardless of their age, menopausal status, grade, stage, and axillary lymph nodes status or tumor size [44, 45]. In this study the positivity of ER and PR were 59 (48.8%) and 54 (44.6%), respectively which is similar to the result of Cherry Bansa [30] in India. He demonstrated ER positive 218 (42.8%) and PR positive 194 (31.8%), but Sohail SK reported positivity for ER and PR receptors in invasive breast carcinoma 70%-80% and 60%-70%, respectively in Pakistan [21], similar results were found by Shafaq Mujtaba in Pakistan [9]. Fouzia Shaikh found 54 (71.1) for ER and the same result for PR [37]. Bernard Seshie in one of the studies found the positivity of ER 53 (32.1%) and PR 42 (25.6%). Jalal Poorolajal from Iran found that 70.8% ER and 66.6% PR positive [8]. All this reveals the heterogeneity of ER and PR positivity among different populations.

Tumor size is one of the key factors of staging BC that can affect the treatment of patients. In this study there was significant correlation of PR with tumor size which shows more positivity in tumors with size of 2–5 cm and ER negative in tumors larger than 5 cm with (*p* = 0.047) and the same result for association of PR but more statistically significant (*p* = 0.021). The study that was done by Bansal & Yadav also found that large size tumor (> 5 cm) are ER negative [30, 38] but Ritu Yadav found that intermediate size of the tumor most of them are PR negative (*p* = 0.040) [38] reveals that growth of the tumor is depended on multiple factors not only ER and PRs but it can be one of the important one. A significant association of ER with histological grade of the tumor found (*p* = 0.014) which show high grade tumors are more ER negative. Same association found statistically significant (*p* = 0.001) by Ritu Yadav, Cherry bansal and other seemlier studies [30, 38, 41, 46] highlights that high grade tumors are negative for ER receptors and show the same relation with PR. Urmila Devi in a similar result found (*p* = 0.71) [43]. In present study there was no significant association between PR and histological grade.

Triple negative (ER, PR and HER2 negative) BC has different clinical and pathological features due to its relatively poor prognosis, aggressive behavior and lack of targeted therapies [19, 47]. In the present study out of 120 patients 23 (19%) were triple negative which is similar to other studies such as Gore with 22% [11] and Cherry with 23.6% triple negative [30]. This type of BR has less benefited of target therapy and more aggressive clinical behavior [48, 49]. 

## Conclusion

Our study demonstrated that, in addition to other salient features, Afghan women diagnosed with BC had a high prevalence of ER and PR negative tumors. Concordant double negativity for ER and PR was also high. Therefore, investigating tumor biology in our population becomes highly relevant in order to develop appropriate diagnostic, therapeutic, and prognostic approaches. Our study showed an association between ER and HER2/neu receptor expression and tumor grade. PR expression did not show a significant association with tumor grade, but there was a significant association with tumor size. Additionally, there was a direct relation between overexpression of HER2 and both tumor size and tumor type.

## Data Availability

The datasets used and/or analyzed during the current study are available from the corresponding author on reasonable request.

## Abbreviations

BC: Breast Cancer
IDC: Invasive Ductal Carcinoma
NOS: No Otherwise Specified
ER: Estrogen Receptor
PR: Progesterone Receptor
Her2/neu: Human Epidermal growth factor Receptor-2/neu
FMIC: French Medical Institute for Mothers and Children
ILMS: Integrated Laboratory Management System
SPSS: Statistical Package for the Social Sciences
IHC: Immunohistochemical
BRCA: Breast Cancer gene
ERBB2: Erythroblast oncogene B2
CIS: Carcinoma in situ
OR: Odds ratio
CDC: Centers for Disease Control and Prevention
CAP: Collage of American Pathologist
ERC: Ethical review committee
MoPH: Ministry of Public Health
MRB: Modified Richardson-Bloom grading system
AKU: Aga Khan University
FISH: Fluorescence in situ hybridization
